# The Inhibition of Autophagy Sensitises Colon Cancer Cells with Wild-Type p53 but Not Mutant p53 to Topotecan Treatment

**DOI:** 10.1371/journal.pone.0045058

**Published:** 2012-09-14

**Authors:** Dan-Dan Li, Ting Sun, Xiao-Qi Wu, Shu-Peng Chen, Rong Deng, Shan Jiang, Gong-Kan Feng, Jing-Xuan Pan, Xiao-Shi Zhang, Yi-Xin Zeng, Xiao-Feng Zhu

**Affiliations:** 1 State Key Laboratory of Oncology in South China, Cancer Center, Sun Yat-sen University, Guangzhou, Guangdong, People’s Republic of China; 2 Department of Pathophysiology, Zhongshan School of Medicine, Sun Yat-sen University, Guangzhou, Guangdong, People’s Republic of China; Istituto Nazionale per le Malattie Infettive, Italy

## Abstract

**Background:**

Topotecan produces DNA damage that induces autophagy in cancer cells. In this study, sensitising topotecan to colon cancer cells with different P53 status via modulation of autophagy was examined.

**Methodology/Principal Findings:**

The DNA damage induced by topotecan treatment resulted in cytoprotective autophagy in colon cancer cells with wild-type p53. However, in cells with mutant p53 or p53 knockout, treatment with topotecan induced autophagy-associated cell death. In wild-type p53 colon cancer cells, topotecan treatment activated p53, upregulated the expression of sestrin 2, induced the phosphorylation of the AMPKα subunit at Thr172, and inhibited the mTORC1 pathway. Furthermore, the inhibition of autophagy enhanced the anti-tumour effect of topotecan treatment in wild-type p53 colon cancer cells but alleviated the anti-tumour effect of topotecan treatment in p53 knockout cells in vivo.

**Conclusions/Significance:**

These results imply that the wild-type p53-dependent induction of cytoprotective autophagy is one of the cellular responses that determines the cellular sensitivity to the DNA-damaging drug topotecan. Therefore, our study provides a potential therapeutic strategy that utilises a combination of DNA-damaging agents and autophagy inhibitors for the treatment of colon cancer with wild-type p53.

## Introduction

Topotecan, a topoisomerase I inhibitor that induces DNA damage, is used to treat colon cancer, ovarian cancer, lung cancer, and advanced cervical cancer [1], [2]. While DNA-damaging agents have been utilised over the past 50 years, the reason that some patients show different sensitivities to a DNA-damaging drug remains unclear. Therefore, insight into the cellular responses triggered by DNA-damaging drugs and the mechanisms that determine drug sensitivity is critical to expand the utility of DNA -damaging drugs for the treatment of cancers.

Autophagy is a catabolic mechanism involved in the recycling and turnover of cytoplasmic components [3], [4]. Autophagy can facilitate cellular survival or death in response to different stress stimuli [5], [6], [7], [8], [9], [10], [11], [12]. Autophagy also plays an essential role in the maintenance of genomic stability [13], [14], [15] by maintaining metabolism and survival during stresses (e.g., DNA damage) to benefit cell survival [16]. Many studies have shown that autophagy is associated with a number of pathological conditions, including cancer[10], infectious diseases, myopathies and neurodegenerative disorders[17], [18], [19]. Because the function of autophagy in cancers is complicated and may have opposing consequences[7], many hypotheses have been proposed regarding the role of autophagy in cancer. One of these hypotheses suggests that the role of autophagy depends on the stage of tumour development[20]. At an early stage of tumour development, genetic evidence firmly indicates that autophagy suppresses tumour initiation. However, compelling data also suggests that established tumour cells, but not initiating tumour cells, require autophagy as a crucial survival pathway at advanced stages of tumour development. Tumours often reside in an environment deprived of nutrients, growth factors, and oxygen. Thus, autophagy is localized to the hypoxic tumour regions that are the most distant from the nutrient-supplying blood vessels where it sustains tumour cell survival. Another hypothesis proposes that autophagy regulates cancer in a cell- and tissue-specific manner [21], [22]. Many cancer cells undergo autophagic cell death after cancer therapies; however, autophagy also protects some cancer cells against anticancer treatments by blocking the apoptotic pathway.

The p53 tumour suppressor is a key molecule in the response to DNA damage. In response to adverse conditions, including genotoxic, hypoxic, and/or oncogenic stress, p53 rapidly undergoes reversible post-translational modifications that facilitate its stabilisation [23]. In the nucleus, active p53 can bind to the promoter regions and transactivate a plethora of target genes involved in cell cycle progression, apoptosis, and/or metabolism [24]. p53 also mediates transcription-independent tumour-suppressing functions outside of the nucleus [25]. For example, cytoplasmic p53 can relocalise to the mitochondria and trigger mitochondrial membrane permeabilisation [26], [27].

In cancer, many links exist between autophagy and p53 that have yet to be fully understood[28]. One study reported that P53 promoted autophagy through AMP-kinase (AMPK) activation and mammalian target of rapamycin (mTOR) inhibition [29]. However, accumulating evidence indicates that the P53 tumour suppressor can modulate autophagy in several manners depending on its subcellular localisation[25]. On one hand, p53 is a transcription factor that responds to cellular stress and transactivates genes such as DRAM, sestrins1 and sestrins2 that induce autophagy or autophagic cell death [30], [31], [32]. However, on the other hand, cytoplasmic but not nuclear p53 can activate mTOR and repress autophagy [33]. Furthermore, p53 can also induce autophagy by regulation of LC3 [34]. However, understanding how these effects are achieved remains elusive.

In the present study, we report that wild-type p53 can activate AMPK, inhibit mTORC1 and promote colon cancer cells survival by enabling cytoprotective autophagy in response to topotecan treatment. Moreover, the inhibition of autophagy sensitises colon cancer cells with wild-type p53 to topotecan treatment. In contrast, the inhibition of autophagy alleviated the anti-tumour effect of topotecan treatment in p53 mutant or knockout colon cancer cells both in vitro and in vivo. Therefore, our study indicates that a combination of DNA-damaging agents and autophagy inhibitors could potentially serve as a novel chemotherapeutic approach for the treatment of colon cancer cells with wild-type p53.

## Results

### Topotecan Treatment Induced Autophagy in Colon Cancer Cell Lines

**Figure 1:**
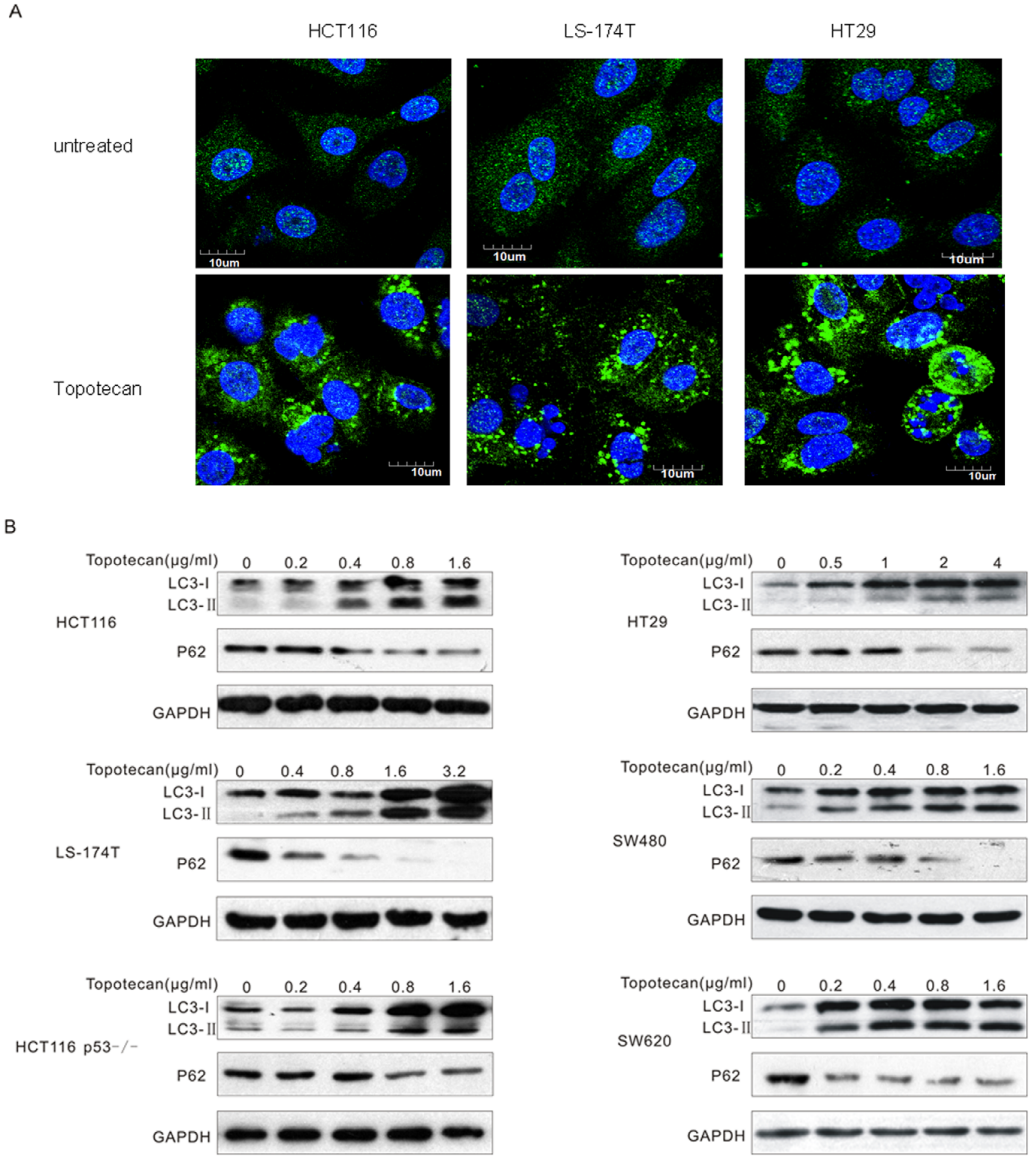
Topotecan treatment triggers autophagy in colon cancer cells. **A.** HCT116, LS-174T and HT29 cells were transfected for 24 h with an expression construct encoding LC3 fused to the yellow fluorescent protein (YFP-LC3). Thereafter, the cells were treated with or without 1 µg/mL topotecan (TPT) for 24 h and visualised under a confocal microscope. **B.** The indicated cells were treated with the indicated concentrations of topotecan for 24 h. The lysates were analysed by immunoblotting with The LC3 and P62 antibodies.

A punctate LC3 staining pattern has been identified as a biological marker of autophagy [35], [36]. To study whether the DNA-damaging agent topotecan could induce autophagy, HCT116, LS-174T and HT29 cells expressing LC3 fused to the yellow fluorescent protein (YFP–LC3) were created. As shown in Fig. 1A, in the untreated cells, the YFP–LC3 was evenly distributed in the cytosol. However, topotecan treatment induced a potent accumulation of YFP-LC3 foci in these cells. LC3 is converted to lipidated LC3 (LC3-II) upon the formation of an autophagosome, and LC3-II migrates faster compared to the nonlipidated LC3-I on an SDS/PAGE gel [37]. We used this difference in migration to further monitor the LC3-II levels after topotecan treatment and found that the appearance of LC3-II was induced at a high level by topotecan treatment in a variety of colon cancer cell lines (Fig. 1B and Fig. S1). Mature auto-lysosomes are subjected to autophagic proteolysis, which leads to a reduced level of the autophagic substrates as well as reduced levels of the autophagosome and auto-lysosome components, such as p62/SQSTM1 [38], [39]. To further confirm that the autophagic process was induced by topotecan treatment, we detected the P62 protein by Western blotting. The results revealed that P62 was degraded after topotecan treatment, which indicates autophagy was induced in these colon cancer cells (Fig. 1B and Fig. S1).

### The Inhibition of Autophagy Increased the Sensitivity of Human Colon Cancer Cells with Wild-type p53 but not Mutant p53 or p53 Knockout Cells to Topotecan Treatment

**Figure 2:**
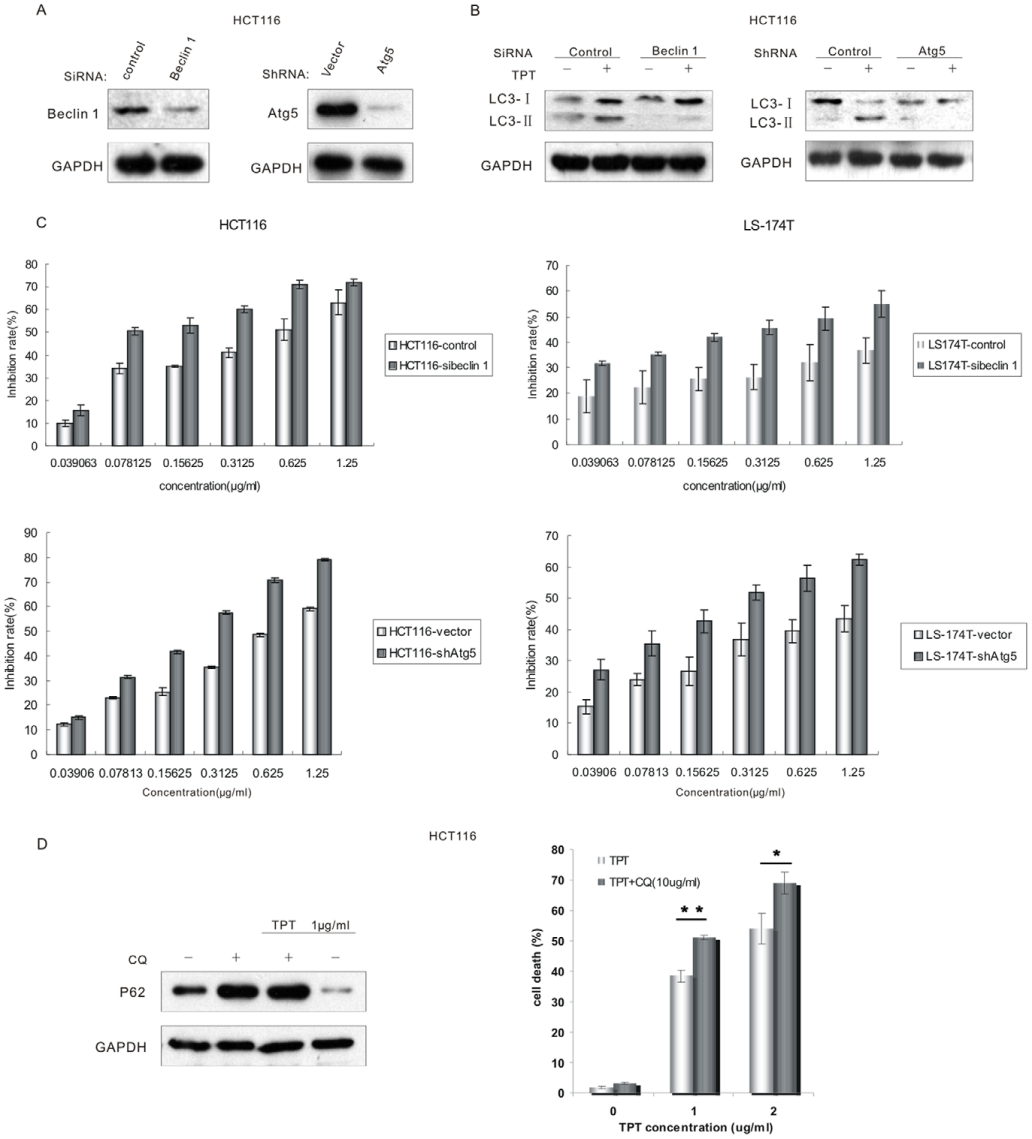
Topotecan treatment induced functional and cytoprotective autophagy in these p53 wild-type cells. **A.** Lysates from HCT116 cells transfected with the beclin 1-siRNA or Atg5- shRNA were analysed by immunoblotting. **B.** An immunoblotting analysis showed no accumulation of LC3-II in autophagy-defective (i.e., beclin 1-siRNA or Atg5-shRNA treated) cells following treatment with 1 µg/mL TPT. **C.** HCT116 and LS-174T control-cells, sibeclin 1-cells, and shAtg5-cells were cultured at 6000 cells per well in a 96-well plate and exposed to different concentrations of topotecan (0.039 to 1.25 µg/mL) for 72 h. The level of growth inhibition was detected using the MTT assay. Data in C are means ± s.d. (*n*  =  3). *P*<0.05, Student’s t test. **D.** HCT116 cells were incubated with 1 µg/mL topotecan and/or 10 µg/mL CQ for 24 h. The lysates were analysed by immunoblotting with the P62 antibodies. HCT116 cells were incubated with the indicated concentrations of topotecan or a combination of topotecan and 10 µg/mL CQ for 24 h. The amount of cell death was quantified using the PI staining assay by flow cytometry. Data in D are means ± s.d. (*n*  =  3). *P<0.05,**P<0.01, Student’s *t* test.

To study the topotecan-induced autophagy in greater detail, RNA interference was used to deplete two known core autophagy proteins, beclin1 and ATG5 (Fig. 2A and Fig. S2A). As shown in Fig. 2B and Fig. S2B, the depletion of beclin 1 or ATG5 by siRNA treatment effectively blocked the accumulation of LC3-II after topotecan treatment, which indicates the inhibition of autophagy. Furthermore, the inhibition of autophagy by the depletion of beclin 1 or ATG5 increased topotecan-induced cell death in the HCT116 and LS-174T cell lines, which are p53 wild-type human colon cancer cells (Fig. 2C). These results were also confirmed by SRB assay (Fig. S3A). These results indicate that topotecan treatment induced functional and cytoprotective autophagy in these p53 wild-type cells. To further confirm our results, we also inhibited the autophagic pathway with pharmacological inhibitors. We found that a co-treatment of topotecan with chloroquine (CQ), an inhibitor of lysosomal function that prevents the completion of autophagy at the final step, effectively blocked the topotecan-induced degradation of P62. Importantly, the inhibition of autophagy by CQ also sensitised HCT116 cells to topotecan-induced cell death (Fig. 2D and Fig. S2C).

**Figure 3:**
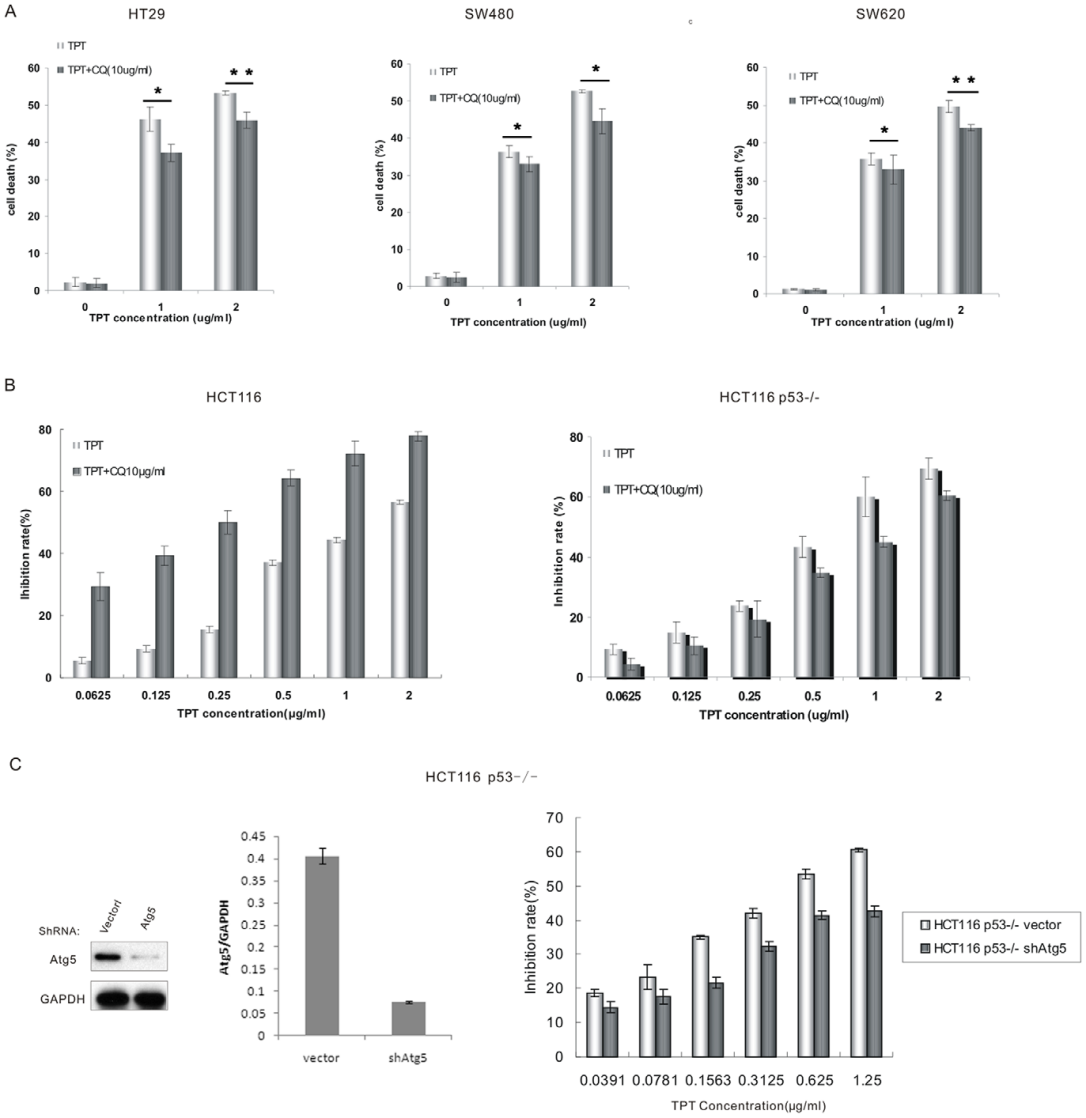
The inhibition of autophagy in p53 mutant cells blocked the cell death induced by topotecan treatment. A. The HT29, SW480, and SW620 cells were incubated with the indicated concentrations of topotecan or a combination of topotecan and 10 µg/mL CQ for 24 h. The amount of cell death was quantified using the PI staining assay by flow cytometry. Data in A are means ± s.d. (*n*  =  3). *P<0.05,**P<0.01, Student’s *t* test **B.** HCT116 p53^+/+^and HCT116 p53^−/−^ cells were cultured at 6000 cells per well in a 96-well plate and exposed to different concentrations of TPT (0.0625 to 2 µg/mL) and/or 10 µg/mL CQ for 72 h. The level of growth inhibition was detected using the MTT assay. **C.** Lysates from HCT116 p53^−/−^ cells transfected with the vector or Atg5 shRNA were analysed by immunoblotting. The MTT assay was used to analyse the level of cell growth inhibition. Data in B,C are means ± s.d. (*n*  =  3). *P*<0.05, Student’s *t* test.

Interestingly, in contrast to the cells with wild-type p53, the inhibition of autophagy by CQ in p53 mutant cells blocked the cell death induced by topotecan treatment (Fig. 3A). Furthermore, in cells where the p53 gene had been somatically knocked out (HCT116 p53^−/−^), the inhibition of autophagy by CQ treatment also blocked topotecan-induced cell death (Fig. 3B). Similarly, the inhibition of autophagy by Atg5 depletion did not sensitise the HCT116 p53−/− cells to topotecan-induced cell death (Fig. 3C and Fig. S3B). In summary, the inhibition of autophagy sensitised colon cancer cells with wild-type p53 to topotecan treatment; however, topotecan-induced cell death was alleviated in the p53 knockout cells upon autophagy inhibition.

### Topotecan-induced Autophagy was Mediated by p53 Through Activation of Sestrin 2 and AMPK in Colon Cancer Cells with Wild-type p53

**Figure 4:**
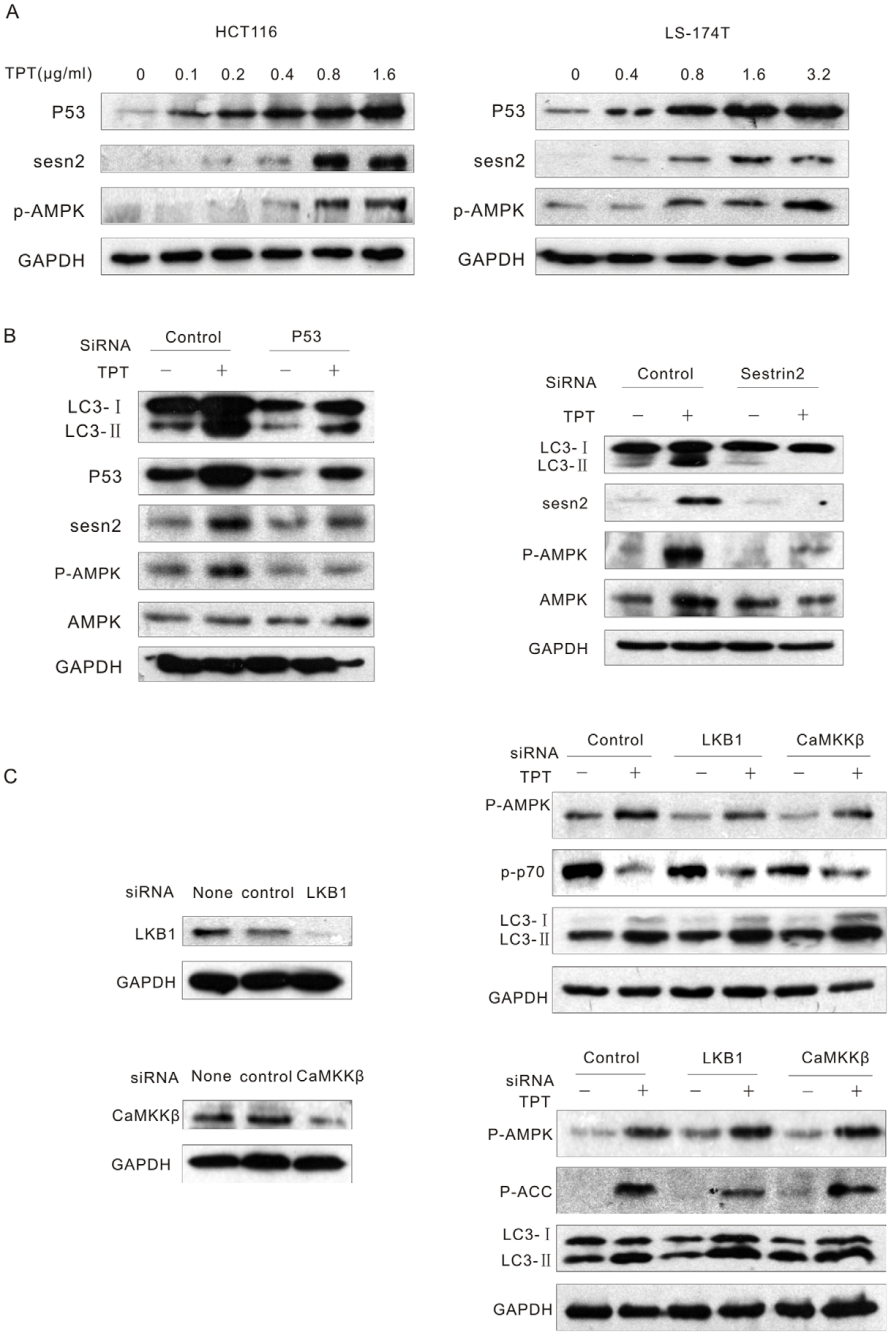
P53 mediates topotecan-induced autophagy through the activation of sestrin 2 and AMPK in colon cancer cells with wild-type p53. **A.** HCT116 and LS-174T cells were treated with various concentrations of TPT for 24 h. The levels of P53, sestrin2 and p-AMPK were analysed by immunoblotting. **B.** HCT116 cells were transfected with p53 or sestrin 2 siRNAs for 24 h, treated with or without 1 µg/mL TPT for an additional 24 h, and the indicated proteins were then analysed by immunoblotting. C. HCT116 cells were transfected with LKB1 or CaMKKβ siRNAs, treated with or without 1 µg/mL TPT for an additional 24 h, and the indicated proteins were detected by immunoblotting.

The P53 tumour suppressor is activated upon multiple types of DNA damage and in turn, inhibits cell proliferation through the induction of specific target genes [40]. Thus, we next attempted to investigate whether P53 and its target genes were involved in topotecan-induced-autophagy. As expected, topotecan treatment stimulated an increase in the level of P53 in a dose-dependent manner in the HCT116 and LS174T cell lines. Furthermore, topotecan treatment also increased the expression of the P53 target gene, sestrin 2. The phosphorylation of AMPK was also notably increased after topotecan exposure (Fig. 4A and Fig. S4A). Additionally, treatment with siRNAs targeting p53 or sestrin 2 effectively abrogated the topotecan-induced AMPK activation and LC3-II accumulation (Fig. 4B and Fig. S4B). These findings suggest that P53 mediates topotecan-induced autophagy through the activation of sestrin 2 and AMPK in colon cancer cells with wild-type p53.

Ca^2+^/calmodulin-dependent kinase β (CaMKKβ) can phosphorylate and activate AMPK in response to increased calcium levels [41], [42]. Studies have also shown that the LKB1 serine-threonine kinase is required for the activation of AMPK in response to stress [43]. AMPK can be phosphorylated by transforming growth factor-β-activated kinase 1 (TAK1), which activates AMPK upon TRAIL treatment [14]. To investigate if these known AMPK activators were involved in this process, the expression of LKB1 and CaMKKβ were knocked down using siRNA. The results demonstrated that even after the efficient depletion of LKB1 and CaMKKβ, neither the topotecan-induced AMPK activation nor the LC3-II accumulation were affected (Fig. 4C and Fig. S4C).

### Topotecan Treatment Induced Autophagy Through the AMPK-mediated Inhibition of mTORC1 in Wild-type p53 Colon Cancer Cells

**Figure 5:**
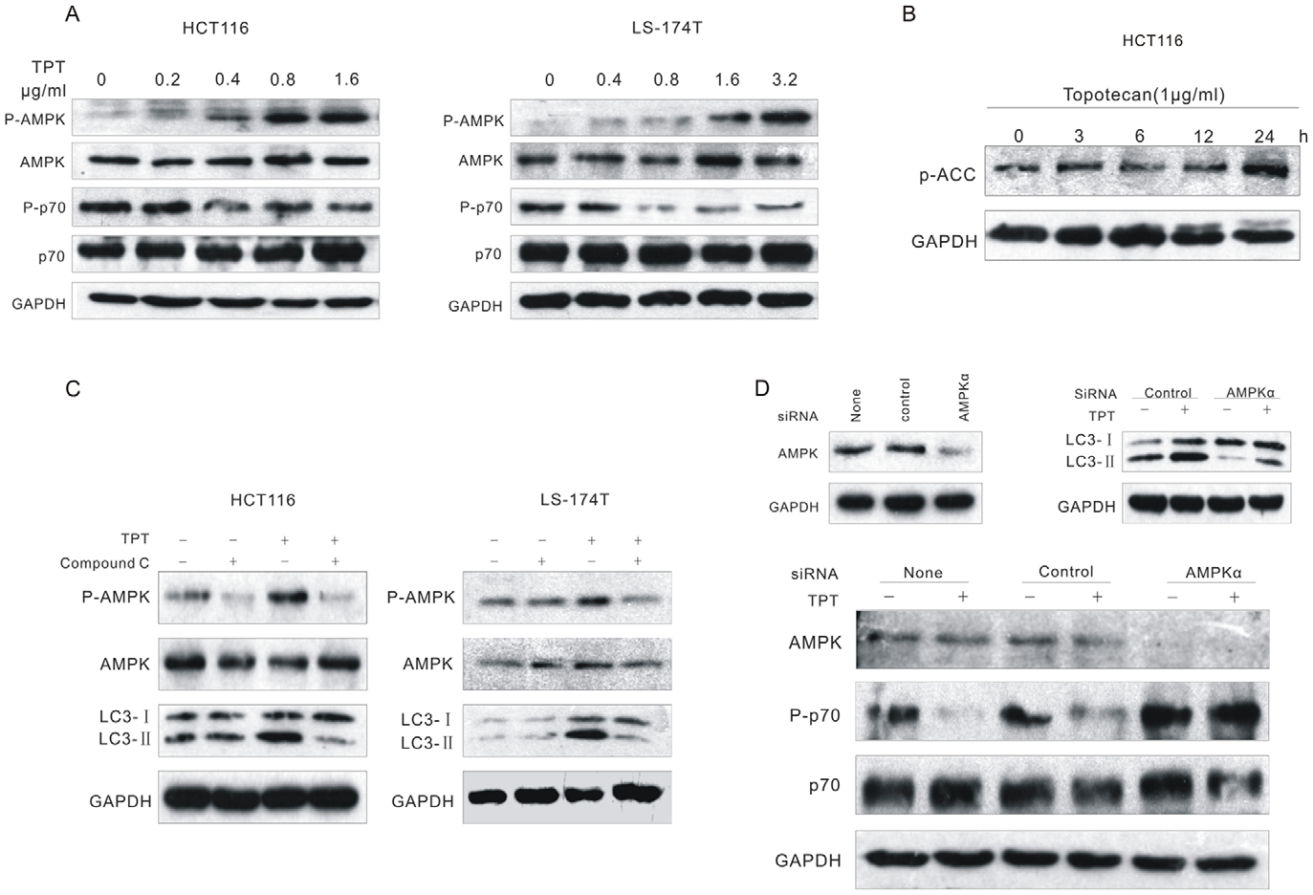
Topotecan treatment induced autophagy through the AMPK-mediated inhibition of mTORC1 in p53 wild-type colon cancer cells. A. HCT116 and LS-174T cells were treated with TPT for 24 h. The expression levels of the indicated proteins were analysed by immunoblotting. **B.** HCT116 cells were treated with 1 µg/mL TPT for the indicated times. The cell lysates were analysed by immunoblotting with phospho-ACC antibodies. **C.** The cells were treated with 1 µg/mL TPT and/or 20 µM compound C for 24 h, and the expression levels of the indicated proteins were analysed by immunoblotting. **D.** HCT116 cells were transfected with control or AMPKα siRNAs for 48 h, treated with or without 1 µg/mL TPT, and the level of the indicated proteins were subsequently analysed by immunoblotting.

AMPK plays a key role in maintaining energy homeostasis and autophagy activation [44]. To examine the effect of topotecan treatment on AMPK activity, HCT116 and LS-174T cells were exposed to topotecan in a dose- and time-dependent manner. Topotecan treatment noteably activated AMPK in the two tested cell lines with wild-type p53, which was shown by the phosphorylation of AMPKα at the active site residue Thr172 (Fig. 5A and Fig. S5A). Also, topotecan treatment induced a rapid and sustained activation of AMPK, which was evident by an increase in the phosphorylation of acetyl-CoA-carboxylase (ACC), a well-known AMPK substrate, at Ser79 (Fig. 5B Fig. S5B). Studies have shown that mTOR can sense changes in the cellular energy state through AMP-activated protein kinase (AMPK), and the inhibition of mTORC1 by AMPK may increase autophagy [45]. Consistent with these results, we found that topotecan treatment inhibited the mTORC1 pathway, which was evident by a reduced phosphorylation of ribosomal protein S6 kinase 1 (p70S6K), a direct mTORC1 substrate (Fig. 5A and Fig. S5A).

To determine whether AMPK activity was essential for topotecan-induced autophagy, we inhibited the activity of AMPK using a pharmacological inhibitor or RNA interference. The pre-treatment of the cancer cells with 20 µM compound C, a potent and selective AMPK inhibitor, effectively prevented topotecan-induced AMPK activation. At the same time, topotecan-induced LC3-II accumulation was also blocked by compound C treatment. These results indicate that autophagy was inhibited upon AMPK inhibition (Fig. 5C and Fig. S5C). The depletion of the catalytic α1-subunit of AMPK using siRNA also confirmed these results. The inhibition of AMPK activity indeed blocked the induction of autophagy, which was evident by the level of LC3-II (Fig. 5D and Fig. S5D). Furthermore, the depletion of AMPKα restored p70S6K activity, which indicated that the inhibition of mTORC1 was relieved (Fig. 5D and Fig. S5D). These data suggest that the autophagy induced by the DNA-damaging agent, topotecan, depends on the AMPK-mediated inhibition of mTORC1.

### The Inhibition of AMPK Activation Increased the Sensitivity of Human Colon Cancer Cells with Wild-type p53 but not Mutant p53 to Topotecan Treatment

**Figure 6:**
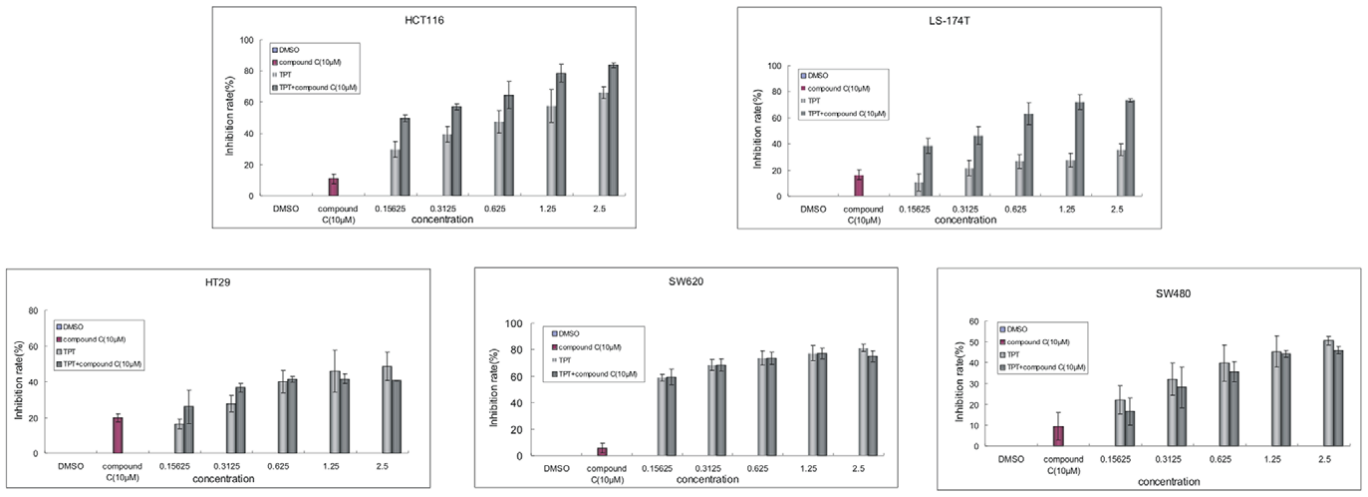
The inhibition of AMPK activation increases the cellular response to topotecan treatment in p53 wild-type but not in p53 mutant colon cancer cells. Various cells were cultured at 6000–8000 cells per well in a 96-well plate and exposed to different concentrations of TPT (0.15 to 2.5 µg/mL) and/or 10 µM compound C for 72 h. The level of growth inhibition was detected using the MTT assay. Data are means ± s.d. (*n*  =  3). *P*<0.05, Student’s *t* test.

In order to confirm whether AMPK activity was essential for cell viability after topotecan treatment in cancer cells with wild-type or mutant p53, human colon cancer cells were treated with various concentrations of topotecan in the presence or absence of the AMPK inhibitor, compound C. As shown in Fig. 6, the combined treatment of topotecan with compound C resulted in an increased level of cytotoxicity in human colon cancer cells with wild-type p53 (HCT116 and LS174-T cell lines). However, compound C treatment did not sensitise cells with mutant p53 (HT29, SW480 and SW620 cell lines) to topotecan treatment. Based on these findings, we concluded that the activation of AMPK serves as a central mediator in the induction of cytoprotective autophagy in response to DNA damage in colon cancer cells containing wild-type p53 but not mutant p53.

### The Anti-tumour Activity of Topotecan in Combination with the Autophagy Inhibitor in a HCT116 Human Colon Cancer Xenograft Model

**Figure 7:**
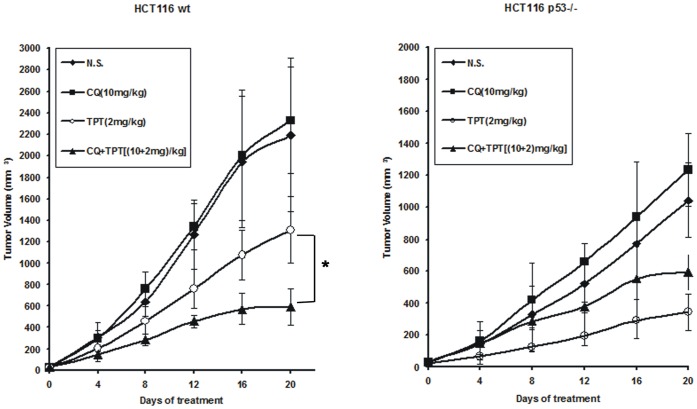
The anti-tumour activity of an autophagy inhibitor in combination with topotecan treatment in a HCT116 human colon cancer xenograft model. Athymic nude mice were injected subcutaneously with 4×10^6^ HCT116 p53^+/+^ or HCT116 p53^−/−^ cancer cells. Six mice were assigned into each of the treatment groups. The tumours were allowed to grow for approximately 5 d to produce an average tumour volume of 40 mm^3^ prior to drug treatment. Topotecan was intraperitoneally administered once every 4 d (2 mg/kg), and CQ was intraperitoneally administered every day (10 mg/kg). The tumour growth was measured every 4 days according to the method described in the “Materials and Methods” section. Results are presented as means ± s.d. (*n*  =  6). *P<0.05, Student’s *t* test.

To explore the in vivo effectiveness of the autophagy inhibitor CQ to sensitise wild-type p53 human colon cancer cells to topotecan treatment, a xenograft model in nude mice was generated. Athymic nude mice were injected subcutaneously with 4×10^6^ HCT116 p53^+/+^ or HCT116 p53^−/−^ cancer cells, and the resulting tumours were allowed to grow for approximately 5 d to produce an average tumour volume of 40 mm^3^ prior to drug treatment. The anti-tumour effects of topotecan, CQ, or a combination treatment of the two drugs were evaluated in HCT116 p53^+/+^ and HCT116 p53^−/−^ xenografts. Topotecan was intraperitoneally administered once every 4 d (2 mg/kg), and CQ was intraperitoneally administered once every day (10 mg/kg). As shown in Fig. 7, a delay in tumour growth was observed with topotecan treatment alone, and the combination of CQ with topotecan treatment increased the anti-tumour effect in the HCT116 p53^+/+^ xenograft model. However, the anti-tumour effect of CQ was not enhanced by the combination treatment with topotecan in the HCT116 p53^−/−^ xenograft model. On the contrary, the combination drug treatment seemed to impair the anti-tumour activity of topotecan in this model.

In addition, the effect of AMPK inhibitor compound C in combination with topotecan on the xenograft model was examined. Having established the effectiveness in vitro for AMPK inhibitor-induced chemosensitization to topotecan in p53 wild-type human colon cancer cells, the xenograft model in nude mice was generated to explore whether AMPK inhibition was involved in chemosensitization to topotecan in vivo. As shown in Fig. S6, compound C played a similar role as CQ in combination with topotecan treatment. Compound C could increase antitumor effect in combination with topotecan in HCT116 p53+/+ but not p53−/− xenograft model.

Therefore, our results suggest that the inhibition of autophagy sensitises colon cancer cells with wild-type p53 to DNA-damaging drugs. Since AMPK serves as a central mediator in the induction of cytoprotective autophagy in response to DNA damage, inhibition of AMPK could also enhance the effect of DNA-damaging drugs to colon cancer cells with wild-type p53.

## Discussion

Autophagy is an evolutionarily conserved lysosomal self-digestion process. The effects of autophagy in tumorigenesis and cancer therapy are paradoxical. It has been reported that autophagy is able to suppress tumorigenesis through keeping genomic stability and elimination of p62. However, utilizing autophagy as a cytoprotective mechanism, tumor cells manage to survive in harsh microenvironment. In response to many anticancer therapies, autophagy is induced as a pro-survival strategy in human cancer cells or a contributing antitumor effect. Therefore, a great deal of effort should be made to augment what decides cytoprotective and cytocidal functions of autophagy, which is essential in targeting at the right autophagic signaling pathways that makes the cancer treatment more promising. Herein, we found that wild-type p53-mediated AMPK activation resulted in cytoprotective autophagy in response to the DNA-damaging drug topotecan in human colon cancer cells. The inhibition of autophagy sensitised colon cancer cells with wild-type p53 to topotecan treatment; however, autophagy inhibition attenuated the anti-tumour effect of topotecan treatment in p53 mutant or knockout colon cancer cells both in vitro and in vivo. Apart from known mechanisms, we suggested that p53 could modulate autophagy and proposed that p53 status could determine cell fate following DNA-damage drug topotecan-induced autophagy and help maintain autophagic homeostasis.

With respect to cancer, many links exist between autophagy and p53. In the present study, we focused on the fate of autophagic cancer cells in response to a DNA damage agent under different p53 status. First, we show that several colon cancer cell lines, including p53 wild-type and p53 mutant/knockout human colon cancer cells, exhibit morphologic and biochemical features characteristic of cells undergoing autophagy following treatment with the DNA-damaging drug topotecan. Furthermore, the blockage of autophagy by the depletion of Atg5 or beclin 1 by RNA interference or by chloroquine treatment, but which alleviated the cytotoxicity of topotecan in cancer cells with mutant p53 or p53 knockout. As we known, topotecan should not only make DNA damage but also arrest cells to G1/S arrest or G2/M arrest depending on distinct cell lines. To explore whether the cell cycle arrest is enough to induce the potentiated cell death after inhibition autophagy, we used vincristine (VCR) as a different type of anti-cancer drugs to confirm our findings. VCR binds to tubulin dimers, inhibiting assembly of microtubule structures. Disruption of the microtubules arrests mitosis in metaphase. In our study, comparing with VCR treatment only, combination with autophagy inhibitor makes no great difference on colon cancer cells whatever the status of the p53 (Fig. S7). The results suggested that topotecan-mediated cell death and cytoprotective autophagy could be associated with DNA damage in colon cancer cells. Our previous studies have shown that autophagy contributes to cell death in CNE2 cells with mutant p53 and Hep3B cells, which are p53 deficient [46], [47], [48]. Although it seems rational to view p53 for its proapoptotic activities, we showed that extension of cell survival through autophagy induction was also an inherent feature of wild-type p53. Such cytoprotective function may be related to the role of wild-type p53 and autophagy in maintaining intracellular metabolic homeostasis. However, some cancer cells with mutant p53 or p53 knockout lost this prosurvival feature of autophagy. Indeed, blockage of autophagy may promote cancer cell death in these conditions. Our findings joined a growing number of examples what is the function of autophagy in cancer therapy and what decides the fate of autophagic cancer cells.

The mechanisms whereby wild-type p53 induces cytoprotective autophagy appear to primarily arise from the transcriptional control of mTOR pathway regulators. Indeed, multiple p53 target genes, such as AMPK, TSC2, and PTEN, are all known negative regulators of mTORC1. Remarkably, two p53 target genes, sestrin1 and sestrin2, have been identified as a critical link between p53 activation and mTORC1 activity [31], [49], [50]. On this basis, we hypothesised that wild-type p53 and its target, sestrin 2, could activate AMPK, inhibit mTORC1 and promote cell survival by enabling a cytoprotective autophagy in response to topotecan. Based on the data shown in this study, this hypothesis appears correct. However, two well-known AMPK activators, LKB1 and CaMKKβ, were not responsible for the topotecan-induced activation of AMPK and the resulting autophagy. As expected, AMPK activation and phospho-p70S6K inhibition were not observed following topotecan treatment in p53 mutant cells (Fig. S8). Furthermore, our data show that the inhibition of AMPK increased cellular sensitivity to topotecan treatment in p53 wild-type cancer cells but not in p53 mutant cells. Our results suggest that in wild-type p53 colon cancer cells, but not mutant p53 cells, the p53 activated-AMPK serves as a central mediator in the induction of cytoprotective autophagy in response to DNA damage.

Collectively,the fate of autophagic cancer cells likely differs among cells with different p53 status. In the case of cancer cells with wild-type p53, p53 promotes the survival factor AMPK. As a result, active AMPK may promote autophagy to protect cancer cells to counteract the cytotoxicity caused by the DNA-damaging agent. Conversely, p53 mutant or loss could not activate AMPK, but might promote other “stress-activated protein kinase” such as c-Jun NH2-terminal kinases, force a high autophagy rates, and eventually cause autophagic cell death. Moreover, we confirm that inhibition of autophagy sensitized colon cancer cells with wild type p53, which in contrast inhibited antitumor effect in the ones with p53 knockout to DNA damage treatment vivo. Therefore, our study provided a potential treatment strategy in which the combination of DNA damage agents with autophagy inhibitor was utilized in cancer cells with wild type p53, however, the combination of DNA damaging drugs and autophagy inducers might be developed into a useful strategy to treat cancers expressing mutant p53 or p53 knockout.

## Materials and Methods

### Ethic Statement

This study and experimental protocols were approved by the Institutional Animal Care and Use Committee of Sun Yat-Sen University with permit number 11002A.

### Cell Culture

HCT116, LS174-T, SW480, SW620 and HT29 cells were grown in DMEM medium supplemented with 10% FBS (heat inactivated at 56°C for 30 min) and the appropriate amounts of penicillin and streptomycin in a 37°C incubator with a humidified 5% CO_2_ atmosphere. The HCT116 p53^−/−^ cell line was kindly provided by Professor Jing-Xuan Pan (Sun-Yat Sen University, China).

### Confocal Microscopy and Indirect Immunofluorescence

The cells were transiently transfected with a yellow fluorescent protein (YFP)-tagged LC3 expression vector using Lipofectamine 2000 (Invitrogen, 11668019). Twenty-four hours after transfection, the cells were treated with topotecan. After a 24 h topotecan treatment, the cells were fixed with 4% paraformaldehyde and examined under a laser-scanning confocal microscope (Olympus, FV-1000).

### RNA Interference

The cells were transfected with the indicated siRNAs at a 100 nM final siRNA concentration using Lipofectamine 2000 according to the manufacturer’s guidelines. The siRNA against human beclin 1 and the control siRNA were purchased from Santa Cruz Biotechnology (sc-29797 and sc-44237). The siRNAs sequences used to target human LKB1, CaMKKβ and AMPKα have been previously reported [14] .

### The Establishment of Atg5 Stable Knockdown Cell Lines

For generating the Atg5 stable knockdown cell lines, the retroviral vector (pSUPER. Puro), which was kindly provided by Professor Musheng Zeng (Sun Yat-sen University, China), was constructed to encode a short hairpin RNA (shRNA) sequence against Atg5. Vesicular stomatitis virus pseudotyped vectors were produced by the transfection of the VSV-GPG producer cell line with 5 µg of DNA using Lipofectamine 2000 in a 6-well plate. The retrovirus-containing supernatants were collected at days 5 to 7 after transfection. Then, HCT116 p53^+/+^ and HCT116 p53^−/−^ cells were infected three times with the retrovirus-containing supernatants, and the stable cell lines were selected with 1 µg/mL puromycin.

### The MTT Assay

The cells were seeded on 96-well plates and incubated for 12 h before exposure to different dilutions and combinations of topotecan (TPT), compound C, and chloroquine (CQ). After a 72 h treatment, 10 µL of MTT (5 mg/mL) was added to each well. The cells were then incubated for 4 more hours at 37°C, and the liquid in the wells was evaporated. DMSO (100 µL) was added to the wells, and the absorbance (570/655 nm) was measured to estimate the cell viability. The level of growth inhibition was calculated, and the IC50 value was determined using Bliss Software.

### The SRB Assay

The cells were seeded on 96-well plates and incubated for 12 h before exposure to different dilutions and combinations of topotecan (TPT), compound C, and chloroquine (CQ). After a 72 h treatment, cell monolayers are fixed with 10% (wt/vol) trichloroacetic acid and stained for 30 min, after which the excess dye is removed by washing repeatedly with 1% (vol/vol) acetic acid. The protein-bound dye is dissolved in 10 mM Tris base solution for OD determination at 510 nm using a microplate reader.

### The PI Staining Assay

The cells were trypsinised with 0.5 mL of 0.25% trypsin for 3 min, collected and resuspended with 1 ml of cold PBS. After adding 0.5 mL of the staining solution (50 µg/mL PI, 100 µg/mL RNaseA, and 0.2% Triton-100), the cells were incubated at 37°C for 30 min in the dark. The levels of cell death were detected by flow cytometry (Beckman Coulter, Fullerton, CA).

### Immunoblot Analysis

Equal amounts of proteins (40–50 µg) were size-fractionated using 6–15% SDS-PAGE gradient gels. The resolved proteins were electrophoretically transferred on to polyvinylidene difluoride membranes and analysed by immunoblotting using an ECL chemiluminescence reagent and XAR film (Kodak, XBT-1) according to the manufacturer’s protocol. Antibodies against glyceraldehyde 3-phosphate dehydrogenase (Santa Cruz, sc-47724), LC3 (Novus Biologicals, NB100–2220), p53 (Upstate, 05–224), sestrin 2 (Abnova, H00083667-M03), beclin 1 (Cell Signaling Technology, 3738), Atg5 (Cell Signaling Technology, 2630), AMPKα (Cell Signaling Technology, 2532), p-AMPKα (Cell Signaling Technology, 2535), p70S6K (Cell Signaling Technology, 2708), p-p70S6K (Cell Signaling Technology, 9234), p-ACC (Cell Signaling Technology, 3661), LKB1 (Cell Signaling Technology, 3530), and CaMKKβ (Santa Cruz, sc-100364) were used at optimised dilutions along with the appropriate HRP-conjugated secondary antibodies. The data were collected from at least three independent experiments.

### Testing the Anti-tumour Activity in Nude Mice

Athymic BALB nu/nu mice (5 weeks of age) were injected subcutaneously with 4×10^6^ HCT116 p53^+/+^ or HCT116 p53^−/−^ cancer cells. The resulting tumours were allowed to grow for approximately 5 d to produce an average tumour volume of 40 mm^3^ prior to drug treatment. The treatment groups consisted of six mice within the following four treatment groups: NS, CQ (10 mg/kg), topotecan (2 mg/kg), and CQ plus topotecan. Intraperitoneal injections of topotecan were given once every 4 d, and intraperitoneal injections of CQ were given once a day. The tumours were also measured every 4 d, and the tumour volumes were calculated according the formula (π/6×length×width^2^).

### Statistical Analyses

Data were compared by Student’s *t* tests, as appropriate. All *p* values were two tailed. *p* values < 0.05 were considered as statistically significant for all experiments. Data were treated with SPSS 13.0.

## Supporting Information

Figure S1 **The density analysis for blotting bands, quantitated by Image J. FigS1 corresponds to** **Fig. 1B** **.**
(TIF)

Figure S2 **The density analysis for blotting bands, quantitated by Image J. Fig. S2A, B, C correspond to** **Fig. 2** **A, B, D.**
(TIF)

Figure S3 **Autophagy plays different role in colon cancer cells according to the status of p53 after topotecan treatment.** A and B. HCT116 and HCT116 p53−/− (control or shAtg5) cells were cultured at 6000 cells per well in a 96-well plate and exposed to different concentrations of topotecan (0.0625 to 2 µg/mL) for 72 h. The level of growth inhibition was detected using the SRB assay. Data are means ± s.d. (*n*  =  3). *P*<0.05, Student’s t test.
(TIF)

Figure S4 **The density analysis for blotting bands, quantitated by Image J. Fig. S4A, B, C correspond to** **Fig. 4A, B, C** **.**
(TIF)

Figure S5 **The density analysis for blotting bands, quantitated by Image J. Fig. S5A, B, C correspond to** **Fig. 5A,B,C** **.**
(TIF)

Figure S6 **The anti-tumour activity of an AMPK inhibitor in combination with topotecan treatment in a HCT116 human colon cancer xenograft model.** Athymic nude mice were injected subcutaneously with 4×10^6^ HCT116 p53+/+ or HCT116 p53−/− cancer cells. Six mice were assigned into each of the treatment groups. The tumours were allowed to grow for approximately 5 d to produce an average tumour volume of 40 mm^3^ prior to drug treatment. Topotecan was intraperitoneally administered once every 4 d (2 mg/kg), and compound C was intraperitoneally administered every day (2 mg/kg). The tumour growth was measured every 4 days according to the method described in the “Materials and Methods” section. Results are presented as means ± s.d. *(n*  =  6). *P<0.05, Student’s *t* test.
(TIF)

Figure S7 **Autophagy inhibition or not had no effect on cell death induced by VCR treatment, regardless of the status of p53.** HCT116 and HCT116 p53−/− cells were cultured at 6000 cells per well in a 96-well plate and exposed to different concentrations of VCR (0.0078 to 0.25 µg/mL) with or without CQ or compound C for 72 h. The level of growth inhibition was detected using the SRB assay. Data are means ± s.d. (*n*  =  3). *P>*0.05, Student’s t test.
(TIF)

Figure S8 **The activation of AMPK is not involved in topotecan-induced autophagy in p53 mutant colon cancer cells.** HT29, SW620 and HCT116 p53−/− cells were treated with various concentrations of TPT for 24 h, and the expression levels of the indicated proteins were determined by immunoblotting.
(TIF)
