# Supplementary figures and images for: The Inhibition of Autophagy Sensitises Colon Cancer Cells with Wild-Type p53 but Not Mutant p53 to Topotecan Treatment
Source: PLoS One. 2012 Sep 14;7(9):e45058. doi: 10.1371/journal.pone.0045058 (PMC3443203; doi:10.1371/journal.pone.0045058)

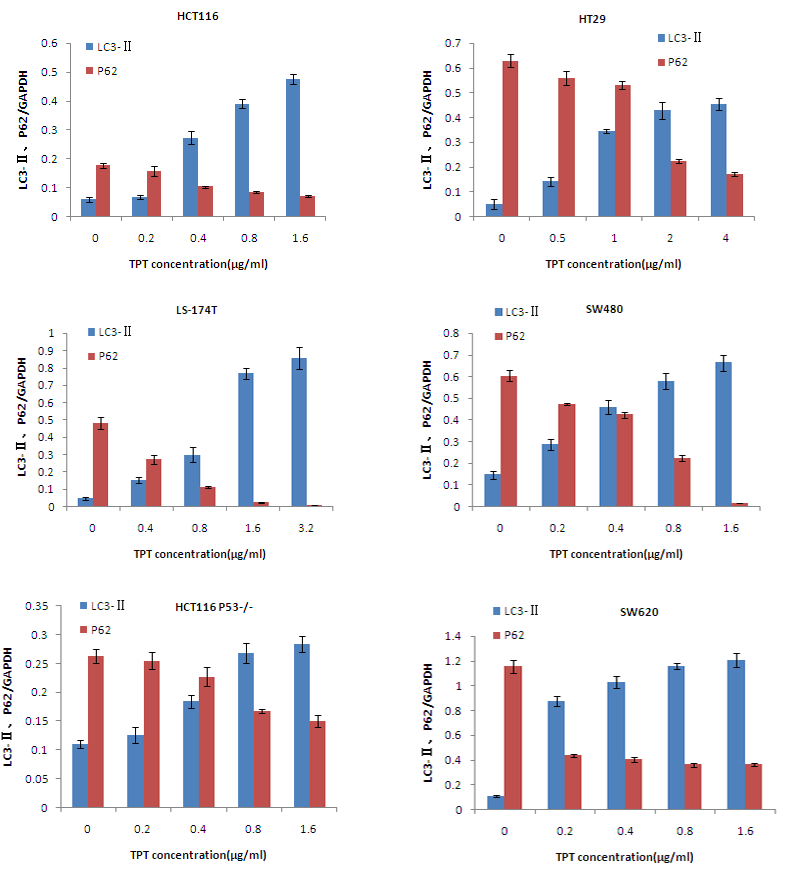

Supplement: Figure S1 — The density analysis for blotting bands, quantitated by Image J. FigS1 corresponds to Fig. 1B . (TIF) [file pone.0045058.s001.tif]

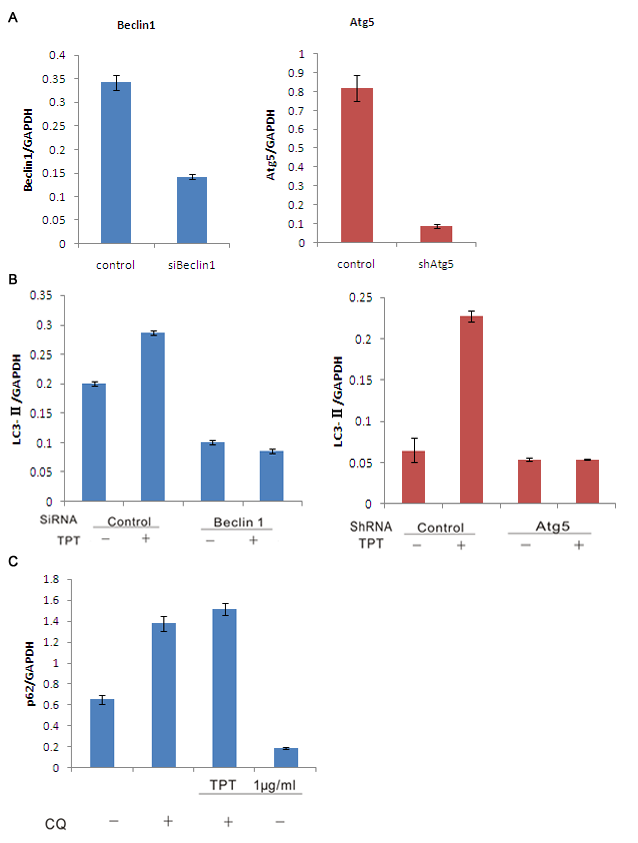

Supplement: Figure S2 — The density analysis for blotting bands, quantitated by Image J. Fig. S2A, B, C correspond to Fig. 2 A, B, D. (TIF) [file pone.0045058.s002.tif]

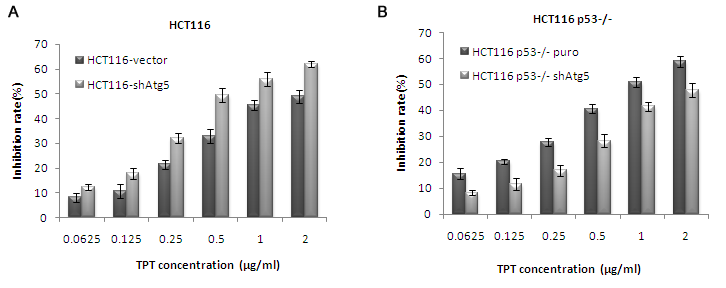

Supplement: Figure S3 — Autophagy plays different role in colon cancer cells according to the status of p53 after topotecan treatment. A and B. HCT116 and HCT116 p53−/− (control or shAtg5) cells were cultured at 6000 cells per well in a 96-well plate and exposed to different concentrations of topotecan (0.0625 to 2 µg/mL) for 72 h. The level of growth inhibition was detected using the SRB assay. Data are means ± s.d. (n = 3). P<0.05, Student’s t test. (TIF) [file pone.0045058.s003.tif]

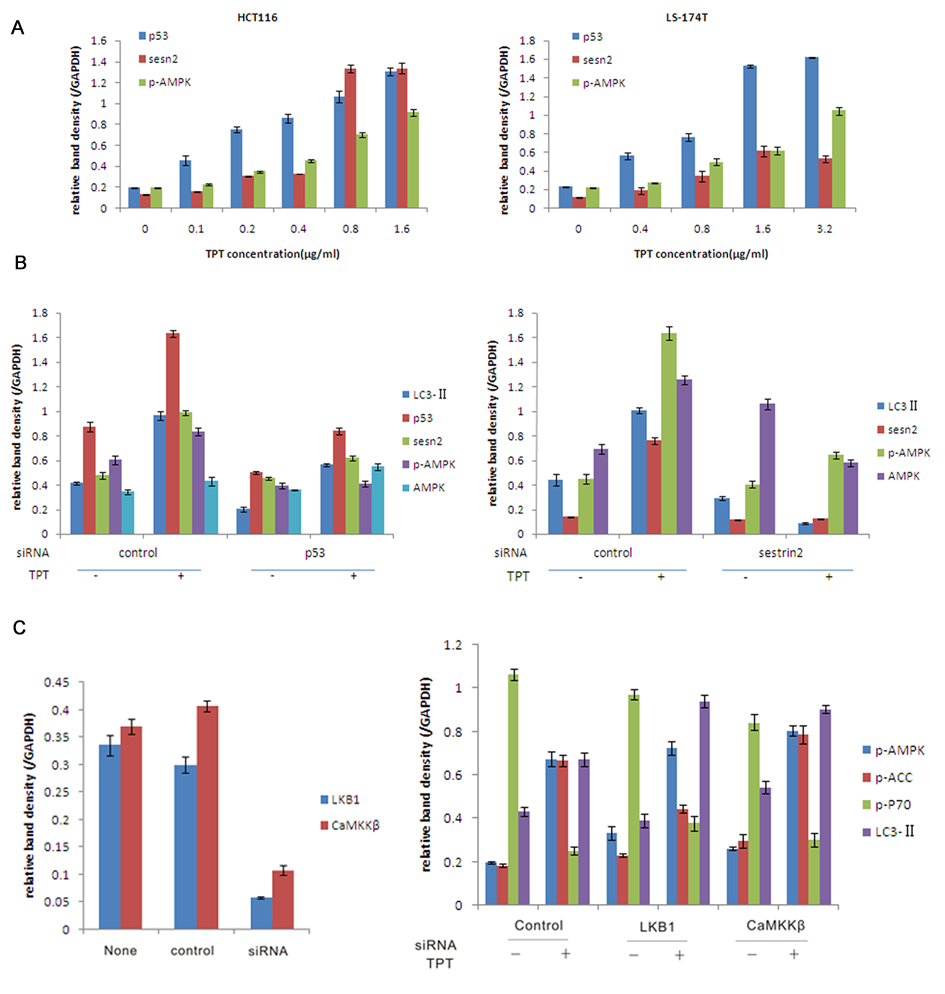

Supplement: Figure S4 — The density analysis for blotting bands, quantitated by Image J. Fig. S4A, B, C correspond to Fig. 4A, B, C . (TIF) [file pone.0045058.s004.tif]

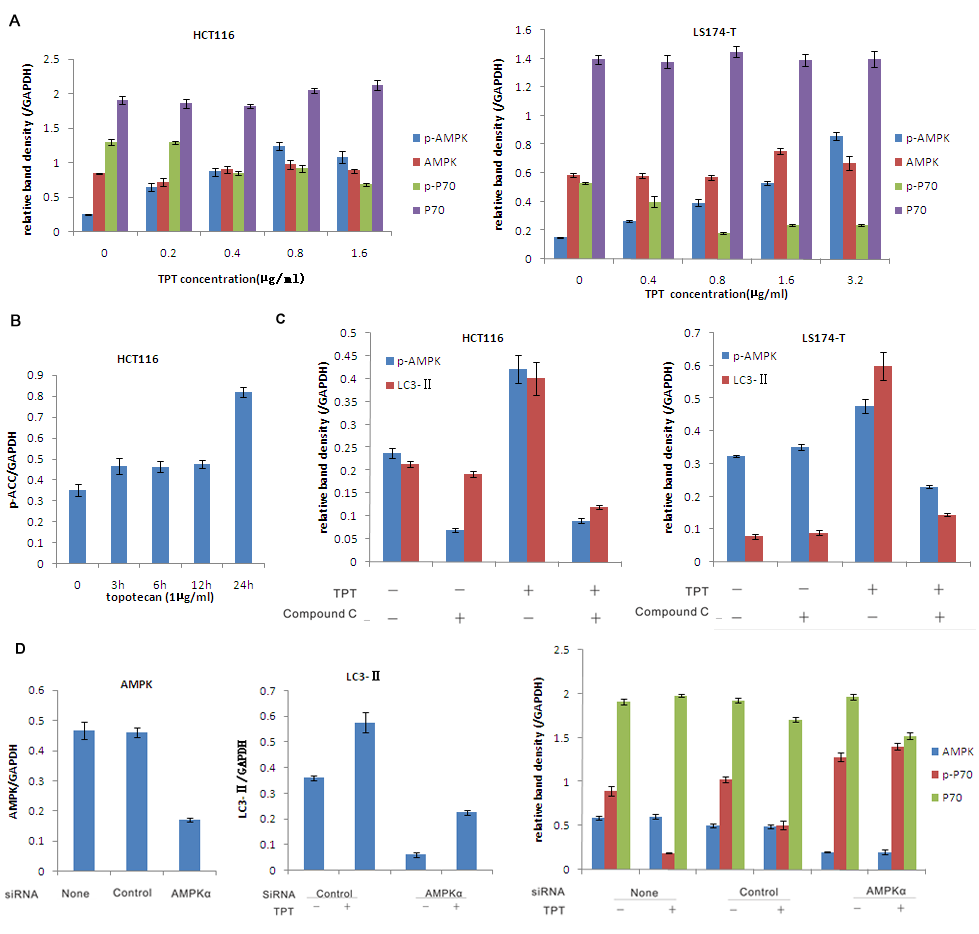

Supplement: Figure S5 — The density analysis for blotting bands, quantitated by Image J. Fig. S5A, B, C correspond to Fig. 5A,B,C . (TIF) [file pone.0045058.s005.tif]

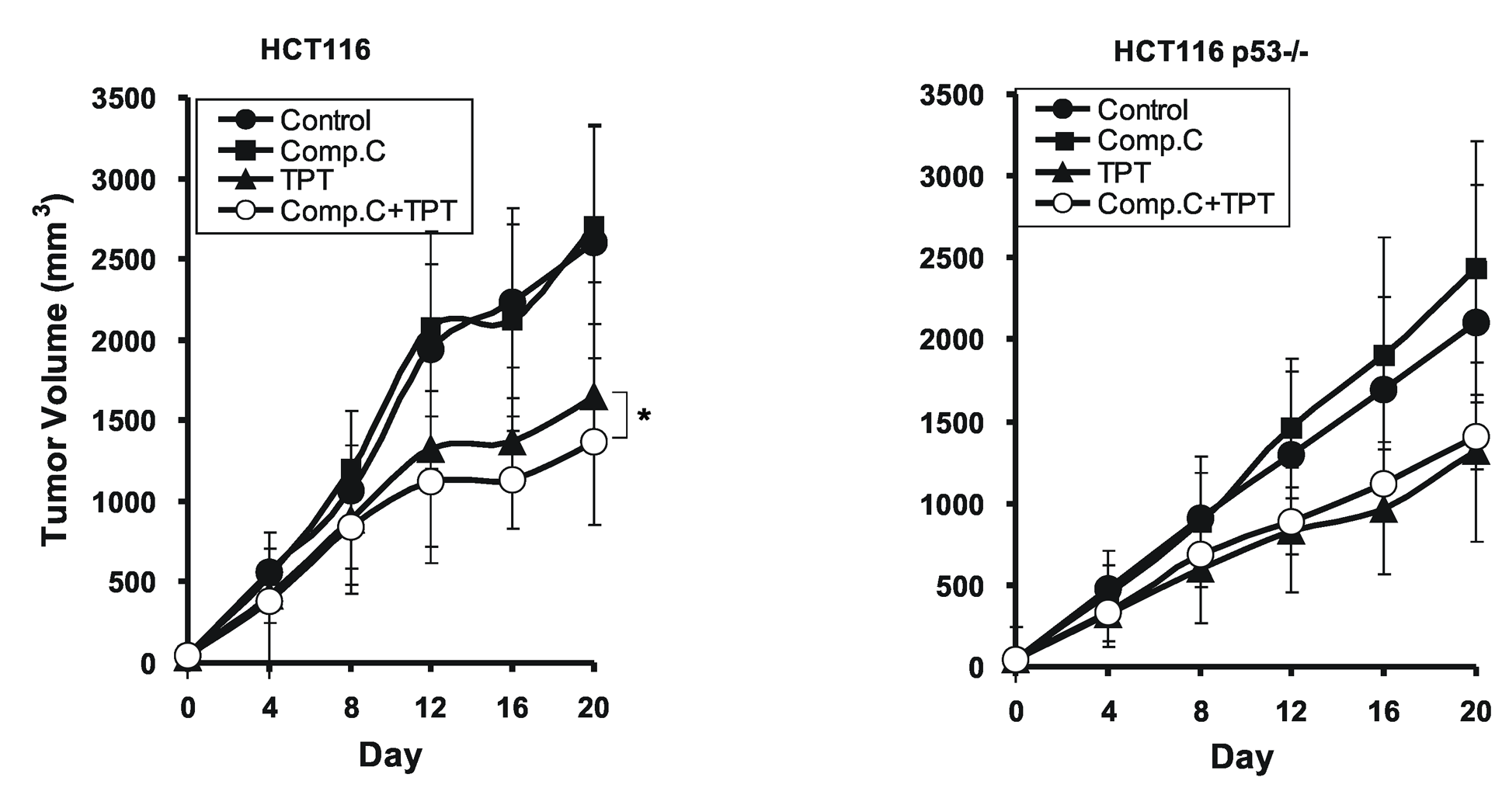

Supplement: Figure S6 — The anti-tumour activity of an AMPK inhibitor in combination with topotecan treatment in a HCT116 human colon cancer xenograft model. Athymic nude mice were injected subcutaneously with 4×10^6^ HCT116 p53+/+ or HCT116 p53−/− cancer cells. Six mice were assigned into each of the treatment groups. The tumours were allowed to grow for approximately 5 d to produce an average tumour volume of 40 mm3 prior to drug treatment. Topotecan was intraperitoneally administered once every 4 d (2 mg/kg), and compound C was intraperitoneally administered every day (2 mg/kg). The tumour growth was measured every 4 days according to the method described in the “Materials and Methods” section. Results are presented as means ± s.d. (n = 6). *P<0.05, Student’s t test. (TIF) [file pone.0045058.s006.tif]

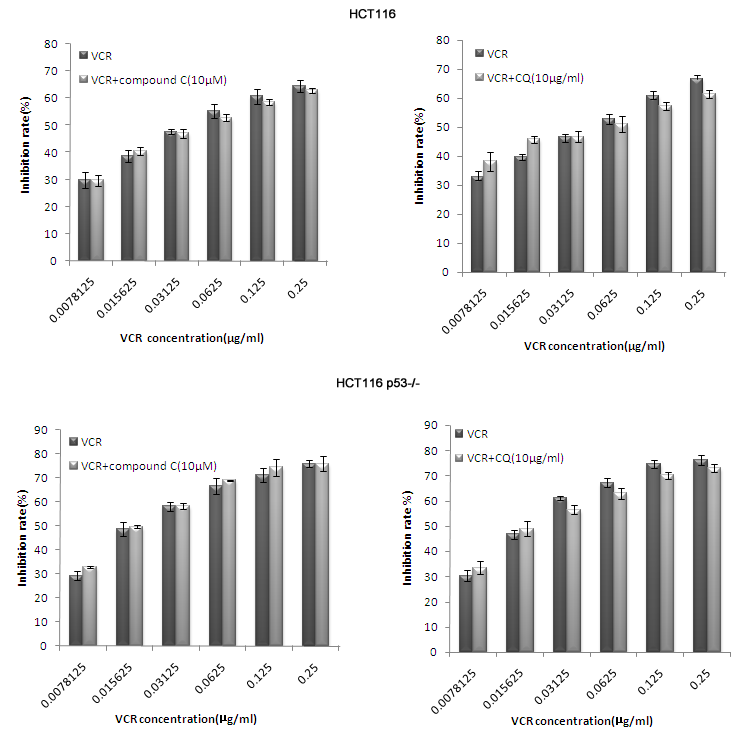

Supplement: Figure S7 — Autophagy inhibition or not had no effect on cell death induced by VCR treatment, regardless of the status of p53. HCT116 and HCT116 p53−/− cells were cultured at 6000 cells per well in a 96-well plate and exposed to different concentrations of VCR (0.0078 to 0.25 µg/mL) with or without CQ or compound C for 72 h. The level of growth inhibition was detected using the SRB assay. Data are means ± s.d. (n = 3). P>0.05, Student’s t test. (TIF) [file pone.0045058.s007.tif]

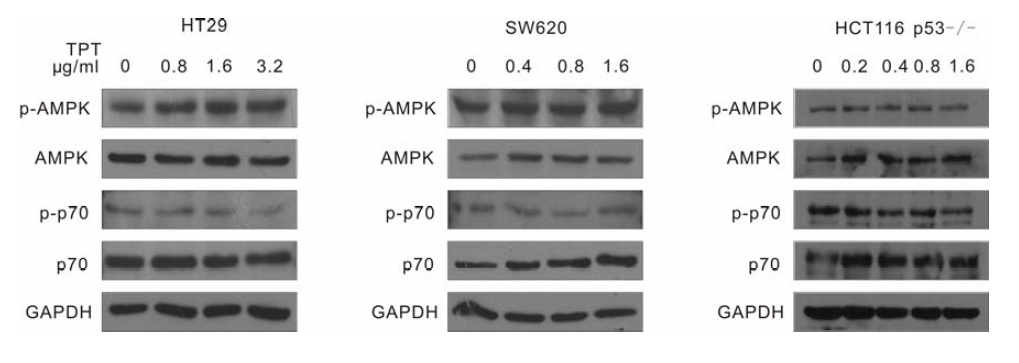

Supplement: Figure S8 — The activation of AMPK is not involved in topotecan-induced autophagy in p53 mutant colon cancer cells. HT29, SW620 and HCT116 p53−/− cells were treated with various concentrations of TPT for 24 h, and the expression levels of the indicated proteins were determined by immunoblotting. (TIF) [file pone.0045058.s008.tif]
